# Anxiety and depression among patients with chronic sciatica

**DOI:** 10.1192/j.eurpsy.2024.724

**Published:** 2024-08-27

**Authors:** A. Feki, I. Sellami, H. Bejaoui, A. Abbes, Z. Gassara, S. Ben jemaa, M. Ezzeddine, M. H. Kallel, H. Fourati, R. Akrout, S. Baklouti

**Affiliations:** ^1^Rheumatology; ^2^occupational medicine, Hedi chaker hospital; ^3^Physical Medicine and Functional Rehabilitation, Habib Bourguiba Hospital, Sfax, Tunisia

## Abstract

**Introduction:**

Spinal radicular syndromes are currently a significant healthcare concern in society. A common manifestation of these syndromes is sciatic pain, characterized by severe pain radiating along the course of the sciatic nerve. In many patients, chronic pain can lead to psychological problems.

**Objectives:**

The aim of this study was to assess the frequency of anxiety and depression disorders in patients with sciatica and their impact on functional capacity.

**Methods:**

We conducted a cross-sectional study, including patients suffering from documented common sciatic pain evolving for more than 3 months. The study was conducted in a rheumatology department over a period of 3 years. We used the Hospital Anxiety and Depression Scale (HADS) questionnaire, supplemented with information about the study group, pain location, and patients’ occupations. Additionally, the Oswestry Disability Index (ODI) and the Visual Analog Scale (VAS) were applied.

**Results:**

The study included 104 patients (71 women and 33 men, with a male-to-female sex ratio of 0.46). The mean age of our patients ranged from 23 to 74 years. The most frequent etiology of sciatic pain was a herniated disc, followed by lumbar spinal stenosis and spondylolisthesis. The root path was L5 in 74 cases and S1 in 30 cases. The average duration of sciatic pain was 6.4 months. The mean Oswestry score was 25 (ranging from 15 to 38). The mean VAS score was 7.4 (ranging from 4 to 9). The mean Work Ability Index (WAI) was 25.2 (ranging from 15 to 38).

Depression was noted in 53 patients (50.9%) with a mean HADS depression score of 10.8 (ranging from 4 to 16). Anxiety was noted in 8 patients (7.6%) with a mean HADS anxiety score of 6.40 (ranging from 3 to 16). In univariate analysis, anxiety was associated with the low educational level of patients and with the duration of sciatic pain (p < 0.05). There was a significant association between depression and anxiety (p = 0.000). However, there was no relationship between these psychiatric disorders and functional status (p > 0.05).

**Conclusions:**

Among patients with sciatic pain, there is a high prevalence of psychiatric disorders, including anxiety and depression. Regular screening for these disorders should be conducted by healthcare providers.

**Disclosure of Interest:**

None Declared

